# Acute Tryptophan Depletion Moja-De: A Method to Study Central Nervous Serotonin Function in Children and Adolescents

**DOI:** 10.3389/fpsyt.2019.01007

**Published:** 2020-03-06

**Authors:** Richard M. Stewart, Janice W. Y. Wong, Simone Mahfouda, Hugo A. E. Morandini, Pradeep Rao, Kevin C. Runions, Florian D. Zepf

**Affiliations:** ^1^Centre & Discipline of Child and Adolescent Psychiatry, Psychosomatics and Psychotherapy, Faculty of Health and Medical Sciences, The University of Western Australia, Perth, WA, Australia; ^2^Brain and Behaviour, Telethon Kids Institute, Perth, WA, Australia; ^3^Specialised Child and Adolescent Mental Health Services, Department of Health, Perth, WA, Australia; ^4^School of Psychological Sciences, Faculty of Science, The University of Western Australia, Perth, WA, Australia; ^5^Community Child and Adolescent Mental Health Services, Department of Health, Perth, WA, Australia; ^6^Department of Child and Adolescent Psychiatry, Psychosomatic Medicine and Psychotherapy, Jena University Hospital, Friedrich Schiller University, Jena, Germany

**Keywords:** acute tryptophan depletion, ATD, Moja-De, central nervous 5-HT, serotonin challenge procedure, ATD Moja-De in child and adolescent populations, attention deficit hyperactivity disorder

## Abstract

Serotonin (5-HT) is widely implicated as a key neurotransmitter relevant to a range of psychiatric disorders and psychological processes. The role of central nervous 5-HT function underlying these processes can be examined through serotonergic challenge methodologies. Acute tryptophan depletion (ATD) is a key challenge method whereby a diminished dietary intake of tryptophan—the amino acid precursor to brain 5-HT synthesis—results in temporary diminished central nervous 5-HT synthesis. While this particular methodology has been used in adult populations, it was only recently that modifications were made to enable the use of ATD in child and adolescent populations. Additionally, the Moja-De modification of the ATD challenge methodology has demonstrated benefits over other ATD techniques used previously. The aim of this protocol paper is to describe the ATD Moja-De methodology in detail, its benefits, as well as studies that have been conducted to validate the procedure in child and adolescent samples. The ATD Moja-De protocol provides a potential methodology for investigating the role of central nervous 5-HT *via* manipulation of brain tryptophan availability in human psychopathology from a developmental viewpoint.

## Introduction

Serotonin (5-HT) is a neurotransmitter that is involved in a variety of psychiatric disorders, such as depressive disorders, anxiety disorders, and attention deficit hyperactivity disorder (ADHD). ADHD is a highly prevalent psychiatric disorder. Recent research has shown that, apart from changes in central nervous dopaminergic activity as well as other neurotransmitter systems (1) the underlying neurobiology of ADHD as a disorder is also subject to changes in 5-HT neurotransmission (2–9). Research on the role of the amino acid (AA) tryptophan (TRP, the physiological precursor of 5-HT) and related brain 5-HT synthesis) have used acute tryptophan depletion (ATD). ATD is a physiological serotonergic challenge procedure that has been widely used over the last decade to investigate the role of 5-HT in a variety of disorders, in both children and adolescents (10, 11), and adults (9, 12). Additionally, ATD allows investigations into the role of 5-HT on various behavioral and cognitive processes. ATD is a safe, transient method of reducing brain 5-HT synthesis by both reducing the central nervous availability of the 5-HT precursor AA TRP and increasing competition for the active transport mechanism that delivers TRP into the brain. In addition, the AAs administered stimulate protein synthesis in the liver, thereby taking TRP from plasma stores and subsequently contributing to the depletion or diminished availability of TRP across the blood brain barrier (BBB). These effects are temporary and are quickly reversed by resumption of a normal diet with adequate levels of TRP. Historically, several ATD protocols have been used since the technique was first applied in humans in 1977, and these methods have included a varying number and/or amount of AAs (13, 14). Until recently, however, there had been no safe and effective short-term neurochemical challenge procedure available to deplete brain 5-HT synthesis in young people, such as children and adolescents.

The more recent development of a new modification of an ATD procedure allows for this particular ATD protocol to be administered to children and adolescents in a safe and effective manner. This new body weight adapted ATD protocol is named Moja-De (a modification of the ATD protocol by Moja and colleagues), and it indirectly accounts for baseline levels of the 5-HT-precursor AA TRP because baseline TRP was shown to be correlated with body weight (15). This new technique therefore allows investigation of the effects of a short-term decrease in brain 5-HT synthesis in young people.

### Acute Tryptophan Depletion and the Development of the Moja-De Protocol

ATD is based on the premise that the depletion of TRP, the physiological precursor AA of central nervous 5-HT synthesis, leads to depletion of central nervous 5-HT. TRP is an essential AA that mammals cannot synthesize and must be acquired from dietary sources. Dietary TRP can be directly used by the gut microbiota to synthesize ligands, kynurenine *via* the kynurenine pathway or peripheral 5-HT through TRP hydroxylase 1 enzyme in the enterochromaffin cells of the gastric mucosa. Thus, gut microbiota can serve as important modulators of TRP availability (4). Plasma TRP levels are influenced by the balance between the dietary intake of TRP and its removal from the plasma by protein synthesis. Most of the TRP in plasma is bound to albumin. Under normal physiological conditions the free TRP portion accounts for 10-15% of total TRP as shown by more recent research (16). The free TRP portion is available for transport into the central nervous system (CNS). Hence, central nervous TRP availability may be more accurately predicted by free TRP than total (free + protein bound) TRP plasma levels. TRP is transported across the BBB by an active transporter (L1), at which TRP and all other large neutral AAs (LNAAs: e.g., valine, leucine, isoleucine, methionine, phenylalanine, and tyrosine) also compete. Once in the brain, TRP is converted into 5-hydroxytryptophan (5-HTP) by the enzyme TRP hydroxylase 2 (the rate-limiting enzyme of central nervous 5-HT synthesis). 5-HTP is then decarboxylated by the enzyme aromatic acid decarboxylase to 5-HT.

There are three factors that are important in determining the rate of brain 5-HT synthesis, and thus three points at which brain 5-HT synthesis may be influenced. These three factors include: (1) The concentration of free TRP in the plasma, which is dependent on dietary intake and/or restriction of TRP, (2) the amount of free TRP that is able to cross the BBB *via* competition for the LNAA transporter with other AAs, (3) the inhibition of the TRP hydroxylase 2 enzyme, [e.g., by para-chlorophenylalanine (PCPA); a rate limiting enzyme for TRP hydroxylase 2, which therefore impacts on central 5-HT synthesis). Central nervous 5-HT synthesis can be influenced by interfering with any or all of these factors. However, the best ATD results in humans with regards to depletion magnitude were achieved using a combination of dietary restriction (point 1), and *via* methods to reduce competitive binding of TRP *via* the LNAA transporter and across the BBB (point 2). Consequently, the ATD technique uses a combination of a low TRP diet and a TRP-deficient protein load containing large amounts of the other LNAAs to produce maximal central nervous TRP depletion (i.e., the administered AAs compete with endogenous TRP on the uptake into the brain over the BBB). A further mechanism that impacts brain TRP availability in the brain is passive diffusion, in both directions, across the BBB. However, this particular mechanism only contributes to a rather small degree (17–19).

The 7-amino-acid ATD mixture as described by Moja et al. (20), was associated with a marked reduction in plasma TRP levels. This protocol was later modified for administration to young people (coined Moja-De, with “De” being related to the last name of Professor Lothar Demisch who suggested and developed this ATD modification) with a body-weight-adapted dosing scheme, and a lower amount of methionine relative to conventional mixtures. The reduction in methionine was to reduce unwanted side effects, such as vomiting and nausea, that were frequently observed in adult populations using alternative formulations (21).

The ATD Moja-De protocol administers AA within an aqueous suspension, in which the relevant AA quantities are dosed in accordance with the participants’ body weights. The AA quantities of the ATD Moja-De beverage are (dosage per 10 kg of body weight): L-phenylalanine (PHE 1.32 g), L-leucine (LEU 1.32 g), L-isoleucine (ILE 0.84 g), L-methionine (MET 0.5 g), L-valine (VAL 0.96 g), L-threonine (THR 0.6 g), and L-lysine (LYS 0.96 g). The TRP Balanced Control (BAL) beverage contains the same AA quantities with an additional 0.7 g of L-TRP per 10 kg of body weight. The chemical properties and descriptions of the eight AAs used as part of the ATD Moja-De and the BAL protocol are summarized in **Table 1**. **Table 1** additionally shows the AAs that comprise the ATD Moja-De mixture (AAs 1 to 7) as well as the amounts of each AA in grams per 10 kg of body weight, as per the body weight adapted protocol. The final beverage composition is included, including excipients. **Table 1** also shows AAs that comprise the BAL mixture, which differs from the ATD mixture only with the addition of the AA L-TRP. The final beverage composition is also included. Specific manufacturing, packaging, and storage details are also provided.

**Table 1:** Chemical properties and descriptions of the AAs and amounts (grams/10kg body weight) that make up the ATD Moja-De and BAL mixture and a brief description regarding manufacturing, packaging and storage details for the AA.

Amino acid chemical name	(1) L-Phenylalanine (PHE)	(2) L-Leucine (LEU)	(3) L-Isoleucine (ILE)	(4) L-Methionine (MET)	(5) L-Valine (VAL)	(6) L-Threonine (THR)	(7) L-Lysine (LYS)	(8) L-Tryptophan (TRP)
**Chemical Structure**	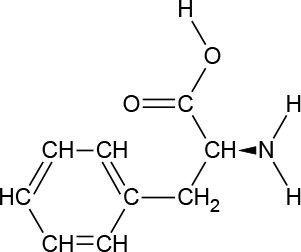	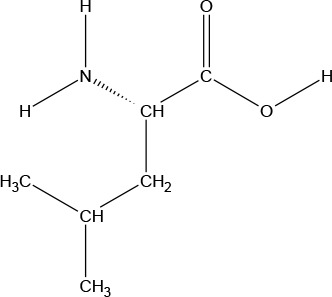	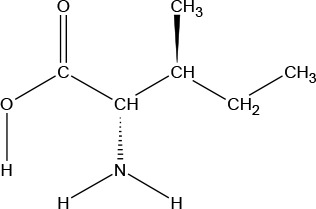	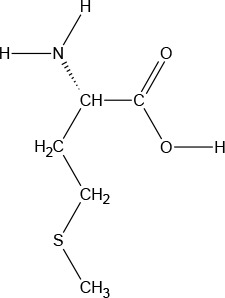	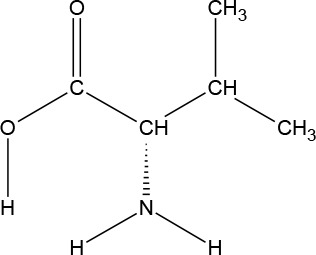	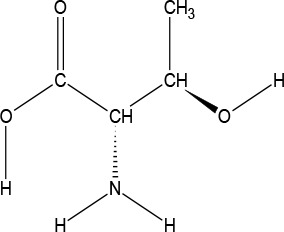	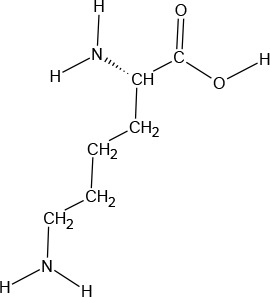	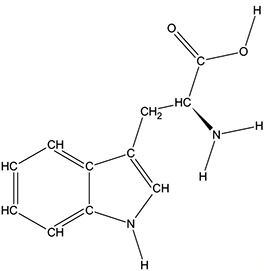
**Molecular formula**	C_9_H_11_NO_2_	C_6_H_13_NO_2_	C_6_H_13_NO_2_	C_5_H_11_NO_2_S	C_5_H_11_NO_2_	C_4_H_9_NO_3_	C_6_H_14_N_2_O_2_	C_11_H_12_N_2_O_2_
**Molecular Weight**	165.19 g/mol	131.17 g/mol	131.17 g/mol	149.21 g/mol	117.15 g/mol	119.12 g/mol	146.19 g/mol	204.23 g/mol
	**(1) L-Phenylalanine (PHE)**	**(2) L-Leucine (LEU)**	**(3) L-Isoleucine (ILE)**	**(4) L-Methionine (MET)**	**(5) L-Valine (VAL)**	**(6) L-Threonine (THR)**	**(7) L-Lysine (LYS)**	**(8) L-Tryptophan (TRP)**
**ATD** (acute tryptophan depletion beverage)→ **amino acids 1-7**	Yes	Yes	Yes	Yes	Yes	Yes	Yes	No
**BAL** (TRP balanced control beverage)→ **amino acids 1-8**	Yes	Yes	Yes	Yes	Yes	Yes	Yes	Yes
**Weight adapted ATD/BAL protocol**	**1.32 g/10 kg** of body weight	**1.32 g/10 kg** of body weight	**0.84 g/10 kg** of body weight	**0.5 g/10 kg** of body weight	**0.96 g/10 kg** of body weight	**0.6 g/10 kg** of body weight	**0.96 g/10 kg** of body weight	***BAL only* 0.7 g/10 kg** of body weight
**Weight/80 kg child or adolescent**	10.6 g/80 kg subject*	10.6 g/80 kg subject*	6.7 g/80 kg subject*	4.0 g/80 kg subject*	7.7 g/80 kg subject*	4.8 g/80 kg subject*	7.7 g/80 kg subject*	***BAL only*** 5.6 g/80 kg subject*
**Formulation**→ *all amino acids are made up to 200 mL with* SyrSpend^®^	10.6 g/200 mL suspension	10.6 g/200 mL suspension	6.7 g/200 mL suspension	4.0 g/200 mL suspension	7.7 g/200 mL suspension	4.8 g/200 mL suspension	7.7 g/200 mL suspension	***BAL only*** 5.6 g/200 mL
	The ATD beverage is made up of the 7 AA (for the ATD aqueous suspension) amounts, and 8 AA (for the BAL aqueous suspension) amounts listed above/80 kg subject* to 200 mL with SyrSpend^®^ SF (Purified Water, Modified Food Starch, Sodium Citrate, Citric Acid, Malic Acid, Sodium Benzoate, Sucralose, Simethicone, and Cherry Flavor). Each subject receives a proportional amount of ATD AAs according to the individual body weight. All body weight adapted dosing steps can be delivered in a beverage of proportional volume of 200 mL of water within an aqueous suspension depending on body weight (see Tab. 6). For example, a 40 kg subject can receive 100 mL with the respective AAs related to the individual body weight.							
**Manufacture process**	An example of a possible manufacture process for the ATD/BAL protocols include the AAs being manufactured and purified through a series of proprietary processing steps. As an example for such processing steps, in a study currently underway (22) these steps have been validated and performed in accordance with GMP under license at: PCCA USA, 9901 South Wilcrest Drive, Houston, TX 77099.							
**ATD/BAL preparation protocol**	Weigh out sufficient powder of each required AA for the batch size. With the mixer running at high speed add required parts of each of the AA powders in turn until all materials are added to the suspension. Allow to cool and deaerate, then make up to final preparation in a calibrated measure. Mix well then allow to cool and deaerate. Measure 203mL into each bottle and seal with a child-resistant cap. Label and store appropriately.							
**Final dosage form and presentation**	Oral suspension of (1) ATD beverage (without L-TRP) or (2) BAL beverage (with 7.0 g/200 mL L-TRP).							
**Container and packaging**	200mL amber light protected glass bottle with white child-resistant screw cap.							
**Storage and handling**	Store at 2–8°C.							
**Stability**	28 days when stored at 2–8°C.							

### Amino Acids Used in the Moja-De ATD Mixture:

#### L-Phenylalanine

l-Phenylalanine (PHE) is an essential aromatic AA in humans (provided by the diet/ood). PHE plays a key role in the biosynthesis of other AAs, and is important in the structure and function of many proteins and enzymes. PHE is converted into tyrosine, which is used in the biosynthesis of dopamine and norepinephrine neurotransmitters. PHE is an odorless white crystalline powder, with a slightly bitter taste and a pH (1% aqueous solution) of 5.4 to 6. It has a water solubility of 26900 mg/L at 25°C.

#### Leucine

Leucine (LEU) is one of nine essential AAs in humans (provided by the diet/food). LEU is important for protein synthesis and many metabolic functions. LEU contributes to regulation of blood-sugar levels; growth and repair of muscle and bone tissue; growth hormone production; and wound healing. LEU is available in many foods and diets, and deficiency is rare. LEU is an odorless white crystalline powder, with a water solubility of 21,500 mg/L at 25°C.

#### L-Isoleucine

l-Isoleucine (ILE) is one of nine essential AAs in humans (present in dietary proteins). L-ILE is a branched-chain aliphatic AA found in many proteins, and it is an isomer of LEUCINE. ILE has diverse physiological functions, such as assisting wound healing, detoxification of nitrogenous wastes, stimulating immune function, and promoting secretion of several hormones. Necessary for hemoglobin formation and regulating blood sugar and energy levels, ILE is concentrated in muscle tissues in humans. ILE is an odorless white crystalline powder, with a bitter taste and a water solubility of 34,400 mg/L at 25°C.

#### L-Methionine

l-Methionine (MET) is a further example of nine essential AAs in humans (provided by the diet/food), MET is required for growth and tissue repair. A sulphur-containing AA, MET improves the tone and pliability of skin, hair, and strengthens nails. Involved in many detoxifying processes, sulphur provided by MET protects cells from pollutants, slows cell aging, and is essential for absorption and bio-availability of selenium and zinc. MET chelates heavy metals, such as lead and mercury, aiding their excretion. It also acts as a lipotropic agent, and prevents excess fat buildup in the liver. MET is a white crystalline powder with a faint odor and sulphurous taste. It has a pH of (1% aqueous solution) of 5.6 to 6.1, and a water solubility of 56,600 mg/L at 25°C.

#### L-Valine

l-Valine (VAL) is an aliphatic and extremely hydrophobic essential AA in humans related to LEU and found in many proteins, mostly in the interior of globular proteins helping to determine three-dimensional structure. A glycogenic AA, VAL maintains mental vigor, muscle coordination, and emotional calm. VAL is obtained from soy, cheese, fish, meats, and vegetables, and VAL supplements are used for muscle growth, tissue repair, and energy. VAL is a white crystalline powder, and has a water solubility of 58,500 mg/L at 25°C.

#### L-Threonine

l-Threonine (THR) is an essential AA in humans (provided by food) and is an important residue of many proteins, such as tooth enamel, collagen, and elastin. It is an important AA for the nervous system, and also plays an important role in porphyrin and fat metabolism and prevents fat buildup in the liver. THR is a white crystalline powder, and has a water solubility of 97,000 mg/L at 25°C.

#### L-Lysine

l-Lysine (LYS) is one of nine essential AAs in humans required for growth and tissue repair, and is supplied by many foods and diets, especially red meats, fish, and dairy products. LYS is a colorless crystalline powder with a sweet/bitter taste. It has a water solubility of 1,000,000 mg/L at 25°C.

#### L-Tryptophan

l-Tryptophan (TRP) is the least plentiful of all 22 AAs and an essential AA in humans (provided by the diet/food), TRP is found in most proteins and a precursor of 5-HT. TRP is converted to 5-hydroxy-tryptophan (5-HTP), which in turn is converted to 5-HT, a neurotransmitter essential in regulating appetite, sleep, mood, and pain. TRP is present in dairy products, meats, brown rice, fish, and soybeans. TRP is an odorless white to slightly yellowish-white crystalline powder, with a slightly bitter taste. It has a water solubility of 13,400 mg/L at 25°C. A 1% solution in water has a pH of 5.5 to 7.

In the administration of the ATD Moja-De Protocol, the aqueous suspension is often administered to young people following an overnight protein fast. The protein fast decreases the dietary intake of TRP, and the LNAAs in the beverage compete with endogenous TRP for uptake into the CNS over the BBB. This subsequently leads to decreased substrate availability for central nervous 5-HT synthesis for a short period of time (approx. 5–7 h). In addition, the administered AAs stimulate protein synthesis in the liver, which takes additional TRP from plasma stores and also contributes to the depletion. The BAL beverage consists of all eight AAs, and causes no significant alteration in brain 5-HT synthesis, as supported by research conducted in rodents (23).

## Validation of the Atd Moja-De/Bal Body Weight-Adapted Protocol

### Animal Models

There are two animal model studies that have successfully validated the ATD Moja-De/BAL body weight adapted protocol (23, 24). Biskup & colleagues (23) validated a refined body weight adapted ATD protocol called Moja-De in two strains of mice, while Sanchez & colleagues (24) examined the neurochemical effects of three developed formulas (ATD Moja-De for 5-HT, phenylalanine/tyrosine depletion or PTD for dopamine (DA) and a combined monoamine depletion mixture or CMD) on brain 5-HT and DA function in mice. Both studies concluded that the Moja-De protocol lowered brain TRP and significantly decreased central nervous 5-HT synthesis.

### Human Validation Studies

There are eight individual study sample populations that have used the ATD Moja-De protocol across healthy and clinical populations of adults and adolescents, and these have generated other associated research papers (see **Table 2**). Given Moja-De’s very likely changed side effect profile compared to other ATD mixtures (which is presumably related to a lower concentration of methionine and the administration of a body weight adjusted ATD protocol and amount of AAs, as well as an overall reduced total amount of AA’s), it is the only ATD protocol so far that has been used in children and adolescents. Across these studies, the ATD Moja-De protocol has been well tolerated overall, with limited side effects/adverse events, and very few drop-outs in studies. In addition, the body-weight-adjusted ATD protocol has been proven to successfully lead to TRP depletion and a decreased CNS of 5-HT, which has enabled Moja-De to be a valid tool to explore the central nervous serotonergic system and related aspects of TRP synthesis. Many of the studies using the Moja-De ATD modification focused on ADHD or ADHD-related symptoms. The following table summarises the findings so far.

**Table 2:** Summary of studies employing the ATD Moja-De Protocol in healthy and clinical populations (young people and adults).

**Title**	**Summary**	**Subjects**
Zepf (25)	This thesis focused on aspects of reactive aggression in young patients with ADHD and Moja-De ATD (aspects related to reactive aggression and Moja-De ATD were published in publications 1A and 1C as listed below). This thesis also gives descriptive information about other variables (heart rate, mood, verbal declarative memory, attentional performance, etc.) that were obtained in this sample but were not part of the thesis topic. These other variables were analyzed later (refer to articles 1C to 1I).	Final sample N = 22. N = 4 were excluded post-hoc after having successfully completed the ATD and BAL challenge protocols because of having received previous neuroleptic medications. N = 5 refused to drink AA mixtures (one of these patients was part of the N = 4 patients who had received neuroleptic medications). N = 2 refused to participate in the remaining study shortly after beverage intake.
Stadler et al. (26)	This study used the ATD Moja-De protocol to study the impact of ATD on aggression in children and adolescents with ADHD. The results indicated that children and adolescents with ADHD behaved more aggressively after ATD when compared to BAL as assessed using a point subtraction aggression game (a psychological task to assess reactive aggression/impulsivity).	The data of N = 22 patients of the above-mentioned sample were analyzed.
**1B**. Zepf et al. (27)	This study examined the effects of ATD on reactive aggression as assessed with a Point Subtraction Aggression Game (PSAG). ATD had a significant effect on increased aggressive behavior with which low-grade impulsive patients responded. High-grade impulsive patients were not affected by ATD.	The data of N = 22 patients of the abovementioned sample were analyzed.
**1C**. Zepf et al. (5)	This study explored the effects of ATD and the reduction of brain 5-HT synthesis on behavioral inhibition in passive avoidance learning assessed in a computerized go/no-go task.	The data of N = 22 patients of the above-mentioned sample were analyzed.
**1D**. Zepf et al. (28)	This study looked at differences in reaction times in the above-mentioned PSAG with regard to the presence of the CBCL dysregulation profile (previously known as the CBCL Paediatric Bipolar Disorder Profile, CBCL-PBD). Comparing those 6 patients with the highest and clinically significant CBCL-PBD scores versus those 6 patients with the lowest, patients with a high CBCL-PBD score showed a slower reaction time under RTD compared to patients with low CBCL-PBD scores after high provocation.	N = 22 patients of the above-mentioned sample were analyzed.
**1E**. Zepf et al. (29)	Low impulsive patients showed a lower heart rate (HR) compared with placebo (those were also patients behaving more aggressively after ATD administration, see paper number 10). Diminished 5-HT functioning was associated with lowered HR.	N = 16 patients of the above-mentioned sample were analyzed.
**1F**. Zepf et al. (30)	This study looked at mood changes after ATD administration when compared to a control condition. ATD had no clear effect on mood. Low scorers on baseline venturesomeness were more strongly affected by ATD in terms of feelings of inactivity and negative feelings compared to high baseline venture patients.	N = 17 patients of the above-mentioned sample were analyzed.
**1G**. Zepf et al. (31)	This study investigated the effects of ATD on attentional performance in children and adolescents with ADHD. Lapses of attention (LA) and phasic alertness (PA) were assessed within the test battery for attentional performance under depleted and sham-depleted conditions 120 (T1), 220 (T2), and 300 (T3) min after intake of ATD or a balanced control condition (BAL). At T1 there was a significant main effect for ATD, indicating more LA after BAL intake compared to ATD. For T2/T3 there were no such effects. PA was not affected by the factors ATD/BAL and time.	N = 22 patients of the above-mentioned sample were analyzed.
**1H**. Zepf et al. (4)	The aim of this study was focus on the participants’ opponent ratings when participating in the PSAG while subjected to ATD and BAL AA mixtures. Following ATD intake, boys with low aggression showed significantly higher extraversion ratings of their fictitious opponent in the PSAG compared to boys with high aggression compared to the control condition.	N = 22 patients of the above-mentioned sample were analyzed.
**1I**. Zepf et al. (6)	The aim of this study was to explore the effects of ATD Moja-De on memory function in young males with attention deficit hyperactivity disorder (ADHD). Overall, there were no significant effects of ATD administration on verbal declarative memory function.	N = 22 patients of the above-mentioned sample were analyzed.
**2A**. Kötting et al. (11)	This study aimed to analyze the effects of ATD on reactive aggression. Boys were more likely to respond with an increased aggressive response after high provocation under ATD. Girls had a higher relative risk than boys of an increased point subtraction in a point subtraction aggression game under ATD after having received a low provocation.	N = 20 young people aged 9 - 15 years (10 female/10 male) with ADHD.
**2B**. von Polier et al. (32)	Here the focus was to study impact of ATD on physiological arousal in 15 young people with ADHD participating in an aggression-inducing game. ATD was not associated with altered physiological arousal, as indexed by electrodermal activity (EDA). Baseline aggression was negatively correlated with the mean ATD effect on EDA. In accordance with the low arousal theory related to aggressive behavior, subjects with reduced physiological responsiveness/lower electrodermal reactivity to ATD tended to display elevated externalizing behavior.	N = 15 young people aged 9 - 15 years (8 female/7 male) with ADHD (a sub-sample of the group studied in the paper by Kötting et al., 2013, study number 2A in this table).
**3A**. Biskup et al. (10)	Alterations of the default mode network (DMN), a network of several brain areas active during rest, have been described in patients with neuropsychiatric disorders, including ADHD. Male children and adolescents with ADHD and healthy controls were subjected to the ATD Moja-De protocol. Three hours after the challenge intake (ATD or balanced control condition, BAL) resting state fMRI scans were obtained. The data indicated that ATD was possibly beneficial to neural planning of motor activity.	N = 22 males (12 – 17 years); 12 with ADHD and 10 neurotypical controls.
**4A**. Dingerkus et al. (21)	This study investigated the effects of diminished central nervous system 5-HT synthesis on plasma concentrations of relevant AAs using the ATD Moja-De protocol. ATD decreased TRP-influx into the brain and CNS 5-HT synthesis safely and effectively and was well tolerated, allowing it to be used in children and adolescents.	N = 24 healthy subjects aged 21 – 30 (N = 12 males and N = 12 females); within-subject repeated measures design with 2 measurement days per subject.
**4B**. Gaber et al. (33)	This study investigated the effects of the Moja-De ATD protocol on punishment-related behavioral inhibition. The results suggested that neurodietary challenges with ATD Moja-De have no clear detrimental effects on task performance and punishment-related inhibition in healthy adults.	See above (same sample as study 4A in this table).
**5A**. Helmbold et al. (34)	This study investigated the effects of diminished CNS 5-HT synthesis *via* the Moja-De ATD protocol on verbal declarative episodic memory while controlling for the effects of female sex hormones. The results indicated that in young women, verbal short-term memory function was more vulnerable to ATD than consolidative processes.	N = 18 healthy female subjects aged 20 – 31 years; within-subject repeated measures design with 2 measurement days per subject
**5B**. Helmbold et al. (35)	This study investigated punishment-induced inhibition in healthy young women while controlling for the menstrual cycle. Following administration of an ATD/balanced control condition (BAL) challenge, neural activity was recorded during a reward or punishment task using fMRI. The results suggested a serotonergic modulation of neural circuits related to emotion regulation, impulse behavior, and punishment in females.	See above (same sample as study 5A in this table).
**5C**. Helmbold et al. (36)	This study examined the serotonergic modulation of intrinsic functional connectivity (FC) with the default mode network (DMN) as assessed with fMRI, while controlling for the menstrual cycle. The results indicated that ATD compared with balanced control condition (BAL) significantly reduced FC with the DMN in areas of the brain associated with self-referential thinking and enhanced FC in areas associated with cognitive reasoning.	See above (same sample as study 5A in this table).
**6A**. Hildebrand et al. (37)	This study investigated the impact of short-term reductions in central nervous system (CNS) 5-HT and dopamine (DA) synthesis *via* an adapted amino acid mixture (phenylalanine-tyrosine depletion, PTD) on phasic alertness (a specific aspect of attention) in healthy adults. The results support an association between decreased CNS DA synthesis and slower reactions times, in line with previous research.	N = 50 healthy adult subjects (25 females, 26 males), between-subject design (ATD: N = 16, PTD: N = 17, BAL: N = 17).
**7A**. Zimmerman et al. (9)	The study examined the effects of the ATD Moja-De protocol on reactive aggression. Lowered rates of reactive aggression were found in the ADHD group under ATD after low provocation, with controls showing the opposite effect.	N = 40 adult subjects (N = 20 with ADHD and N = 20 healthy controls), within-subject repeated measures design.
**7B**. Grabemann et al. (38)	This study aimed to investigate whether diminished brain 5-HT synthesis *via* ATD can impair the processing of affective prosody (the emotional tone of language) in adults with ADHD. The results indicated that there was no clear evidence that 5-HT was implicated in accurately processing affective prosody.	See above.
**7C**. Mette et al. (12)	The study investigated the effects of ATD and the resulting reduction in CNS 5-HT synthesis on discrimination ability and sustained attention. The results support the contribution of serotonergic neurotransmission to attentional processes.	See above.
**8A**. Demisch et al. (15)	This study examined the relationship between body weight and dose effect of ATD with an aim to standardize the ATD methodology. The results suggested that a body weight adapted ATD-test Moja-De protocol that contained a greater amount of phenylalanine (PHE) at the expense of tyrosine, appeared to be a suitable model for standardization of ATD studies.	N = 14 healthy adult volunteers (N = 7 females and N = 7 males), within-subject repeated design.

## Contraindications and Side Effects

The ATD Moja-De protocol should be administered according to the individual body weight, and the studies above demonstrate safety, in addition to depletion-related efficacy and/or behavioral effect-related efficacy (for example, effects on behavioral parameters like reactive aggressive behaviors). The AAs contained in the ATD and the BAL beverages are derived from a normal diet. The beverages are contraindicated for use in participants with known hypersensitivity to the active substance or any of the excipients, as well as in participants with known changes in, or disorders of AA metabolism and participants with psychotic features. Every use in human populations needs to be carefully evaluated (risk vs. benefits analysis) before the beverages are used.

The known side effects of acute ATD Moja-De are mild and short-lived and include an unpleasant taste, nausea, and/or vomiting (22) and lowered mood (27). These are expected to be transient but nevertheless, clear risk mitigation strategies should be in place, such as the administration of a TRP replacement meal following the ATD Moja-De protocol.

## Discussion

ATD protocols other than Moja-De have been used to investigate central nervous 5-HT function of healthy subjects and across a range of diverse clinical populations, particularly in adults (39). The ATD Moja-De protocol was adapted from the seven AA ATD mixture formulated by Moja et al. to account for the individual’s body weight (20), and the Moja-De AA mixture also contains AA’s with a high affinity to the L-1 transporter at the BBB, thus allowing efficient competitive antagonism with regards to central nervous TRP uptake. This relatively new ATD protocol indirectly accounts for baseline levels of TRP, the precursor for central nervous 5-HT. Additionally, the Moja-De protocol contains a lower amount of methionine (which contains sulphur) and also an overall smaller amount of AA’s compared to other ATD protocols, and this has likely reduces side-effects such as vomiting and nausea (21). Combined, adjusting for body weight, reducing the overall amount of AAs, and reducing methionine levels has led to the development of a safe and effective neurochemical challenge procedure for the use in young people.

Administration of the ATD Moja-De protocol in both animal and human models of neuropsychiatric disorders have consistently demonstrated lowered brain TRP and significantly decreased central nervous 5-HT synthesis. Given this, the ATD Moja-De protocol can be further extended and applied to investigate central nervous 5-HT functioning in related neuropsychiatric disorders as well as behavioral, physiological, and neuropsychological processes in young people. A specific example includes the ATD Moja-De protocol being employed as a method to investigate predictors of treatment response to selective serotonin reuptake inhibitor (SSRI) administration in adolescents with major depressive disorder (22).

## Ethics Statement

This is a methods paper that reviewed a series of studies. Please see each individual paper for the name and affiliation of the respective ethics committee that approved each individual study.

## Author Contributions

FZ conceptualized this manuscript and was significantly involved in the evaluation of the protocol and subsequent studies. FZ, RS, JW, KR, PR, SM, and HM contributed to the writing of this manuscript.

## Conflict of Interest

In the last 10 years, FZ was the recipient of an unrestricted award donated by the American Psychiatric Association (APA), the American Psychiatric Institute for Research and Education (APIRE), and AstraZeneca (Young Minds in Psychiatry Award). FZ has also received research support from the European Union, German Federal Ministry for Economics and Technology, the German Society for Social Paediatrics and Adolescent Medicine, the Paul and Ursula Klein Foundation, the Dr. August Scheidel Foundation, the IZKF fund of the University Hospital of RWTH Aachen University, the Telethon Perth Children’s Hospital Research Fund (TPCHR); the Princess Margaret Foundation, and a travel stipend donated by the GlaxoSmithKline Foundation. FZ was the recipient of an unrestricted educational grant, travel support, and speaker honoraria by Shire Pharmaceuticals, Germany. In addition, FZ has received support from the Raine Foundation for Medical Research (Raine Visiting Professorship), and editorial fees from Co-Action Publishing (Sweden)/Taylor & Francis Publishing (USA).

The remaining authors declare that the research was conducted in the absence of any commercial or financial relationships that could be construed as a potential conflict of interest.
